# Editorial: Peritoneal dialysis: Recent advances and state of the art

**DOI:** 10.3389/fphys.2023.1185810

**Published:** 2023-04-12

**Authors:** John Larkin, Pasqual Barretti, Thyago Proença de Moraes

**Affiliations:** ^1^ Fresenius Medical Care, Global Medical Office, Waltham, MA, United States; ^2^ Botucatu Medical School, São Paulo State University, Universidade do Estado de São Paulo, Botucatu, São Paulo, Brazil; ^3^ Post Graduate Program in Health and Biological Sciences, Pontifícia Universidade Católica do Paraná, Curitiba, Paraná, Brazil

**Keywords:** peritonitis, cardiovasclar disease, peritoneal membrane, technique failure, catheter insertion, nutrition, fluid overload

## Introduction

Peritoneal dialysis (PD) is a widely used treatment for patients with kidney failure/end-stage kidney disease, and a suitable alternative to hemodialysis (HD). Existing for over 40 years (BOEN, 1961), the adoption of PD has varied widely across the world for different reasons (Struijk, 2015; Pecoits-Filho et al., 2020; United States Renal Data System, 2022). The Global Kidney Health Atlas reports that, on average, 38.1 per million population (pmp) of people receive chronic PD treatment, which is considerably lower than the estimated global prevalence of HD, which is 298.4 pmp (Bello et al., 2019).

**FIGURE 1:**
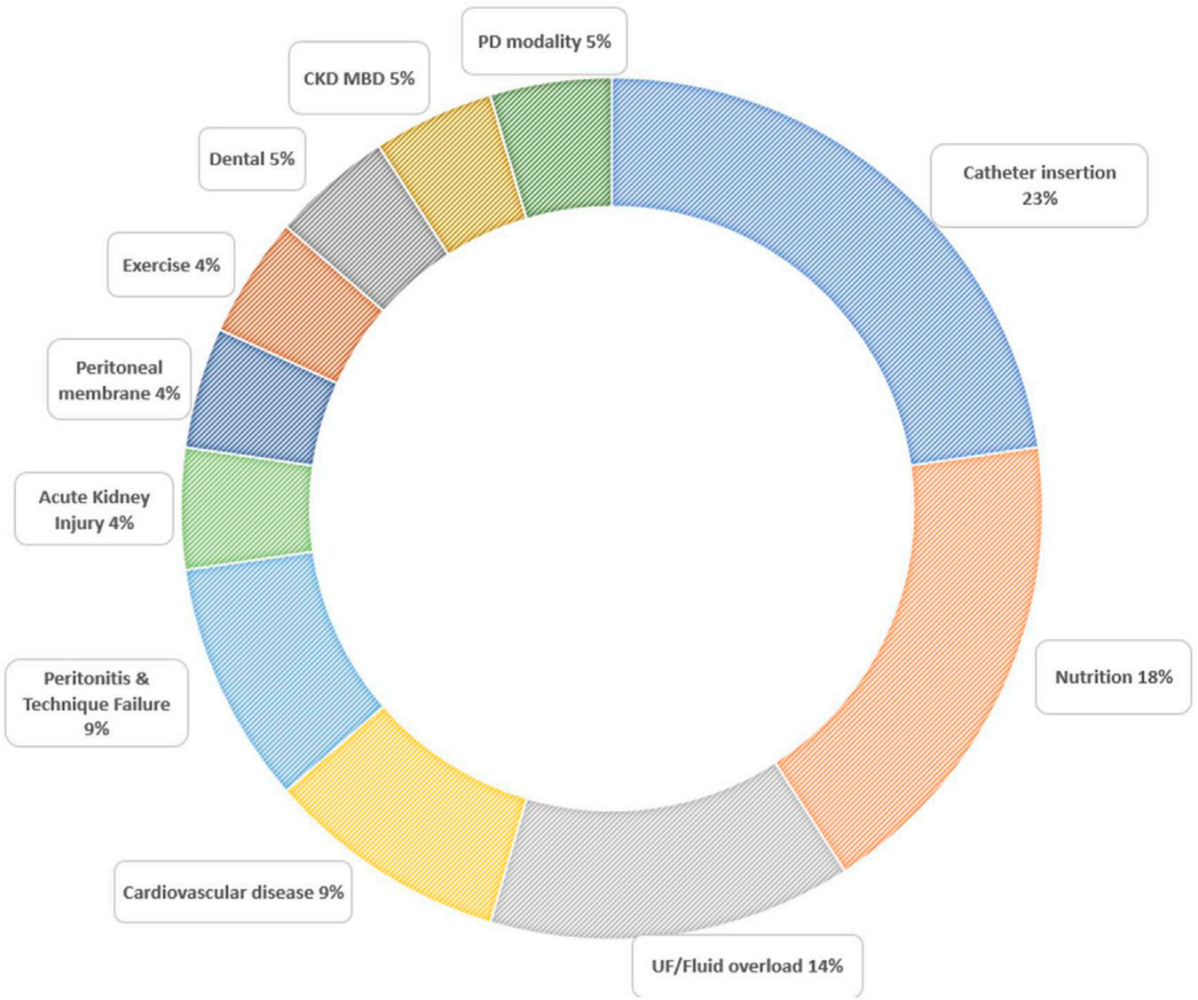
Distribution of PD themes within the Research Topic.

PD has several advantages over HD, including better preservation of residual kidney function, better blood pressure control, and a more flexible and independent treatment schedule that provides greater patient autonomy (Sinnakirouchenan and Holley, 2011; Francois and Bargman, 2014; Brown et al., 2020). PD can also have lower costs for patients and the healthcare system, making it an attractive option for some patients and providers. Despite the potential benefits of PD, there are also challenges associated with the modality including fluid control and solute removal, among other factors (Sinnakirouchenan and Holley, 2011; Brown et al., 2020). This Research Topic highlights 22 contributions from experts who explored several of these challenges and brought new insights learned across four continents. The topic of the contributions varied within this Research Topic (Figure 1), demonstrating the many areas in which improvements and advancements are needed in the delivery of PD and care of patients using the modality. Although we continue to lack important information in the field, these contributions bring a better understanding of the therapy. We invite you to read and enjoy all of them.

## Featured publications

Some topics were more common in this Research Topic, and we’d like to highlight them here. The leading theme was related to problems with catheter insertion, which accounted for almost one out of every four papers published in this Research Topic. PD catheter insertion is a mandatory step in initiating and maintaining chronic PD, and the rates of catheter dysfunction can reach upward to 30% (Szeto et al., 2017). Given this, catheter insertion is always arousing great interest in the PD community. Of these five manuscripts directly related to catheter insertion, two explored different implantation techniques (Jakšić et al.; Yamashita et.), and three evaluated risk factors for catheter failure Chang et al.; Zhao et al.; Hangai et al. Two of these manuscripts add an interesting discussion to the literature about the impact of unplanned PD on outcomes Zhao et al.; Hangai et al.

In second comes manuscripts involving nutrition, totalling four articles Do et al.; Roth-Stefanski et al.; Verger et al.; Guedes et al. Nutrition is a critical aspect of care for patients on PD, as it can affect both short-term and long-term outcomes. These manuscripts shed light on several important aspects related to nutrition in PD including the risk factors for sarcopenia, the diagnosis of protein-energy-wasting, impact of PD prescription on body composition, and an interesting discussion involving inflammation, nutrition, and protein loss.

Other themes included three papers that explored ways to try to optimize ultrafiltration and prevent volume overload in PD (Maggiani-Aguilera et al.; Song et al.; Zhang et al.), two papers that investigated ways to improve detection and prevention cardiovascular disease in the PD population (Kharche et al.; Xia et al.), as well as two papers that investigated risk factors for peritonitis and associated technique failure events (He et al.; Li et al.). Finally, but not less important, other articles of this series investigated associations in CKD-MBD biomarkers and survival (Huang et al.), the use of PD for patients with chronic liver failure and acute kidney injury (Ponce et al.), periodontal disease and alveolar bone loss in PD (Sun et al.), a method to increase physical activity and quality of life in a controlled trial (Zhang et al.), and a review detailing the pathophysiology of peritoneal membrane aging (Krediet).

## Summary

In summary, all the excellent articles published in this Research Topic reinforce the huge variety of needs in the provision care in PD patients, advancements by the scientific community, and the numerous research opportunities to be addressed within the field of PD. Ongoing efforts are needed to improve the quality of care for patients on PD and to increase the availability and utilization of the modality worldwide.
